# Dietary inclusion of raw potato or high‐amylose‐corn resistant starches fed for different durations: impact on phenotypic responses and indicators of gut health of broiler chickens

**DOI:** 10.1002/jsfa.14312

**Published:** 2025-04-29

**Authors:** Iyabo W. Oluseyifunmi, Oluyinka A. Olukosi

**Affiliations:** ^1^ Department of Poultry Science University of Georgia Athens GA USA

**Keywords:** raw potato starch, high‐amylose‐corn starch, growth performance, nutrient transporters, gut health

## Abstract

**BACKGROUND:**

This 21‐day study evaluated the effects of feeding durations of graded levels (RSL) of raw potato starch (RPS) or high amylose corn starch (HCS) on growth performance, energy utilization and intestinal biochemical metrics in broiler chickens. In total, 300 male broiler chicks were randomly assigned to 10 treatments: three RSL (25 and 50 g kg^−1^ of RPS and 35 g kg^−1^ of HCS) and three feeding durations (RSD) (21, 14 or 7 days), plus a corn‐soybean meal control diet.

**RESULTS:**

There were significant effects (*P* < 0.05) of RSL on feed conversion ratio (FCR), apparent ileal digestible energy (AIDE) and nitrogen‐corrected metabolizable energy (AMEn). Birds fed 25 g kg^−1^ RPS had greater FCR than those fed 35 g kg^−1^ HCS. The AIDE and AMEn were greater in birds fed 35 g kg^−1^ HCS, except AIDE at 50 g kg^−1^ RPS and control diets. The RSD × RSL interaction was significant (*P* < 0.05) for jejunal glucagon‐like peptide‐2 (GLP‐2) and RSD for mucin‐2 (MUC‐2) expression. Birds fed 25 g kg^−1^ RPS for 21 and 14 days had higher GLP‐2 expression than those fed for 7 days. MUC‐2 expression was greater for 21 and 14 days of RS feeding than 7 days.

**CONCLUSION:**

The RSL modulated growth performance and nutrient uptake, whereas gene expression was associated with RSD and RSL synergistic effects. © 2025 The Author(s). *Journal of the Science of Food and Agriculture* published by John Wiley & Sons Ltd on behalf of Society of Chemical Industry.

## INTRODUCTION

Resistant starches have been classified as part of complex carbohydrate fractions and considered as dietary fibers.^1^ Therefore, resistant starches (RS), as functional fiber, could be promising for improving gut health in poultry as a result of the production of microbial metabolites such as short‐chain fatty acids (SCFA) when fermented in the caeca.^2^ The metabolites in the digesta can influence the intestinal health by inducing shifts in microbial community composition or by altering the trajectory of the digestion process.^3^, ^4^ The SCFA produced from RS fermentation act by modulating the metabolites in systemic circulation, nutrient digestion and the microbial community in the gut as a result of reduced luminal pH, hence improving the overall intestinal health exhibiting prebiotic effects in the distal gastrointestinal tract (GIT).^5^

In a previous study, we reported that different sources and dietary levels of RS differentially modulated the growth performance, nutrient digestion and utilization, as well as intestinal health, in broiler chicken.^6^ In addition to concentrations and sources of RS, the length of feeding can also influence the effect of RS on growth and gut health metrics.^7^, ^8^ Nofrarías *et al*.^9^ and Qin *et al*.^8^ observed an improvement in colonic mucosal integrity and enhanced villi structure, in pigs and ducks, respectively, when fed diets containing raw potato starch, which depended largely on the starch level and feeding duration. In addition, a duration‐dependent negative impact on the nutrient retention, gut morphology and feed efficiency was reported in broiler chickens fed diets containing high concentration of dietary Hi‐maize resistant starch.^10^

However, only very limited studies have investigated the effects of RS feeding duration in poultry especially in broiler chickens, and the existing results showed high variability as a result of different RS sources and levels, necessitating further investigation. Furthermore, in our previous experiment, increasing RS levels showed variable effects on growth performance, and so it becomes pertinent to investigate whether this could be feeding‐duration dependent. The present study therefore evaluated the effects of duration of feeding (or adaptation lengths) of raw potato starch (RPS) and high amylose corn starch (HCS) on growth performance, nutrient utilization, intestinal morphology, cecal SCFA profile and mRNA expression of nutrient transporters and gut integrity genes in broiler chickens.

## MATERIALS AND METHODS

### Animal ethics statement

The animal experiment was approved by the Institutional Animal Care and Use Committee of the University of Georgia (IACUC number: A2021‐06‐006).

### Birds, diets, housing and experimental design

In total, 300 male broiler chicks (by‐products of the female line) at 1 day old were randomly allocated to 10 treatments in a 3 × 3 + 1 factorial arrangement. The birds were blocked by body weight. The factors were 3 RS levels (RSL), namely 25 or 50 g kg^−1^ of raw potato starch (RPS) and 35 g kg^−1^ of HCS, and three durations (RSD): namely 21, 14 or 7 days, plus a corn‐soybean meal control diet. The feeding durations indicate how long the birds received the RS diets. The birds received the control diet until feeding of the RS diet was initiated in respective treatments. Birds receiving RS diets for 21, 14 or 7 days received the corn‐soybean meal control diets for 0, 7 or 14 days, respectively (Table 1). Each treatment had six replicates and five birds per replicate cage. The birds were housed in metabolism cages in a controlled environment, adhering to the recommended lighting and temperature regimes for Cobb 500 broiler chickens. Feed and water were supplied *ad libitum* for the duration of the experiment. The diets were designed to meet the nutrient and energy requirements for Cobb 500 broiler chickens^11^ and were isocaloric and isonitrogenous. The starter diets were presented in mash form and the grower diets were provided as pellets. The RS used in the current experiment are non‐chemically modified, food‐grade, type‐2 resistant starches, and their starch contents have been reported previously.^6^ The sources of the RS are provided below (Table 2). The feedstuff and chemical compositions of the starter and grower diets are shown in Tables 2 and 3, respectively.

**Table 1:** The treatment arrangement used in the experiment

Feeding duration (RSD), days	Age (days) of birds when the experimental diet was introduced	Number of days the bird received the experimental diets^a^
0 (Control)	–	0
21	0	21
14	7	14
7	14	7

^a^ Three experimental diets were fed: corn‐soybean meal diets with 25 or 50 g kg^−1^ raw potato starch or 35 g kg^−1^ high‐amylose corn starch. The birds in the control treatment received the corn‐soybean meal without any additional source of resistant starch throughout the experiment. The birds in the other treatments (apart from the control) received the corn‐soybean meal control up to the day experimental diets were fed according to the treatment schedule.

**Table 2:** Feedstuff and chemical compositions (g kg^−1^, as fed) of the starter phase (days 0 to 7) diets

Items	Control	Raw potato starch		Hi‐amylose corn starch
Corn	633	606	573	582
Raw potato starch^a^	0	25	50	0
High‐amylose corn starch^b^	0	0	0	35
Soybean meal	323	328	335	330
Soybean oil	9	12	18	19
Dicalcium phosphate	17.5	17.5	17.5	17.5
Limestone	5.9	5.9	5.87	5.87
Sodium bicarbonate	2	2	2	2
Vitamin and trace mineral premix^c^	2.5	2.5	2.5	2.5
dl‐Methionine	1.5	1.6	1.6	1.6
l‐Lysine HCl	1.7	1.7	1.55	1.7
L‐Threonine	0.7	0.6	0.6	0.7
Phytase (Quantum blue)	0.1	0.1	0.1	0.1
Salt	2.8	2.4	2.4	2.4
Total	1000	1000	1000	1000
Calculated nutrients (g kg^−1^ dry matter)				
Crude protein	212	212	212	211
ME (MJ kg^−1^)	12.5	12.5	12.5	12.5
Total P	6.85	6.80	6.75	6.74
Ca	7.96	7.97	7.97	7.96
Non‐phytate P	4.29	4.28	4.27	4.26
Digestible amino acids, g kg^−1^				
Lys	12.60	12.68	12.68	12.67
Met	4.80	4.89	4.88	4.86
Thr	8.64	8.56	8.59	8.62
Met+Cys	8.27	8.34	8.32	8.28
Analyzed nutrient composition (g kg^−1^ dry matter)				
Crude protein	213	208	212	198
GE (MJ kg^−1^)	18.6	18.6	18.7	18.9
Total starch	463	440	452	435

^a^ Raw potato starch used is an RS2 starch sourced from local supplier (Bakers Authority, Maspeth, NY, USA).

^b^ High‐amylose corn starch (Hi‐Maize® 260) was sourced from Ingredion Inc. (Westchester, IL, USA). It is a high‐amylose corn‐based, non‐chemically modified starch.

^c^ Vitamin A, 3527360 IU kg^−1^; vitamin D3, 1 399 921 ICU kg^−1^; vitamin E, 19400 IU kg^−1^ vitamin B12, 8.8 mg kg^−1^; menadione, 1102 mg kg^−1^; riboflavin, 3527 mg kg^−1^; d‐pantothenic acid, 5467 mg kg^−1^; thiamine, 970 mg kg^−1^; niacin, 20 282 mg kg^−1^; vitamin B6, 1455 mg kg^−1^; folic acid, 573 mg kg^−1^; biotin, 79 mg kg^−1^; choline chloride, 771 mg kg^−1^; calcium, 3.20–4.20%; manganese 13.40%; zinc, 10.70%; magnesium, 2.68%; iron, 2.63%; copper, 40 000 ppm; iodine, 1000 ppm; selenium, 400 ppm.

**Table 3:** Ingredients and chemical composition (g kg^−1^, as fed) of the grower phase (days 7–21) diets

Items	Control	Raw potato starch		Hi‐amylose corn starch
Corn	663	637	606	611
Raw potato starch	0	25	50	0
High‐amylose corn starch	0	0	0	35
Soybean meal	295	300	305	302
Soybean oil	8	10	15	19
Dicalcium phosphate	10.5	10.6	10.7	10.7
Limestone	9.2	9.15	9.1	9.1
Sodium bicarbonate	2	2	2	2
Vitamin and trace mineral premix^a^	2.5	2.5	2.5	2.5
dl‐Methionine	1.6	1.6	1.7	1.7
l‐Lysine HCl	1.8	1.7	1.6	1.7
L‐Threonine	0.5	0.5	0.5	0.5
Phytase (Quantum blue)	0.1	0.1	0.1	0.1
Titanium dioxide	3	3	3	3
Salt	2.8	2.4	2.4	2.4
Total	1000	1000	1000	1000
Calculated nutrients (g kg^−1^ dry matter)				
Crude protein	201	201	201	200
ME (MJ kg^−1^)	12.6	12.6	12.6	12.6
Total P	5.3	5.51	5.47	5.47
Ca	7.37	7.39	7.40	7.40
Non‐phytate P	3.03	3.03	3.04	3.04
Digestible amino acids (g kg^−1^)				
Lys	11.92	11.92	11.91	11.91
Met	4.77	4.76	4.84	4.82
Thr	8.00	8.02	8.02	7.98
Met+Cys	8.09	8.06	8.12	8.10
Analyzed nutrient composition (g kg^−1^ dry matter)				
Crude protein	228	226	214	211
GE (MJ kg^−1^)	12.6	12.6	12.6	12.6
Total starch	513	519	474	465
Resistant starch	6	8	12	13

^a^ Vitamin A, 3527360 IU kg^−1^; vitamin D3, 1 399 921 ICU kg^−1^; vitamin E, 19400 IU kg^−1^ vitamin B12, 8.8 mg kg^−1^; menadione, 1102 mg kg^−1^; riboflavin, 3527 mg kg^−1^; d‐pantothenic acid, 5467 mg kg^−1^; thiamine, 970 mg kg^−1^; niacin, 20 282 mg kg^−1^; vitamin B6, 1455 mg kg^−1^; folic acid, 573 mg kg^−1^; biotin, 79 mg kg^−1^; choline chloride, 771 mg kg^−1^; calcium, 3.20–4.20%; manganese 13.40%; zinc, 10.70%; magnesium, 2.68%; iron, 2.63%; copper, 40 000 ppm; iodine, 1000 ppm; selenium, 400 ppm.

### Growth performance measurements

The birds and feeds were weighed on days 0, 7, 14 and 21. The feed intake (FI), body weight gain (BWG), final body weight gain (FBW) and feed conversion ratio (FCR) were determined from days 0 to 21 and corrected for mortality.

### Sample collection

Birds were humanely euthanized using CO₂ asphyxiation on day 21. The ileal digesta (from Meckel's diverticulum to 2 cm proximal to the ileocecal junction) were pooled per cage to calculate ileal nutrient digestibility. The excreta were collected in the evening of day 19 and morning of day 20 (24‐h excreta collection) to evaluate total tract N retention and metabolizable energy. The caecal contents were collected on day 21 for caecal SCFA profile and pH determination.

### Caecal content short‐chain fatty acid profile

Analysis for SCFA followed previously established procedure.^12^ The caecal content was diluted at a ratio of 1 to 3 with deionized water (1 g of digesta and 3 mL of water) and centrifuged for 10 min at 10000 × *g*. Then, 1 mL of the supernatant was combined with 0.2 mL of 25% metaphosphoric acid solution and stored at −20 °C overnight. After thawing and re‐centrifugation, 0.75 mL of the supernatant was mixed with 0.15 mL of an internal standard and 1.8 mL ethyl acetate, then vortexed for 15 s. After settling for 5 min, 1.2 mL of the top layer was pipetted into a glass vial, and the SCFA content was measured using gas chromatography (Shimadzu GC‐2010 Plus; Shimadzu Corporation, Kyoto, Japan).

### Caecal content pH measurement

The caecal content samples pooled from three randomly selected birds per pen were used for pH determination. One gram of sample was mixed with 9 mL of distilled water and vortexed. The pH was subsequently determined using a sterile glass pH electrode with the mixture being stirred with a magnetic stirrer for thorough homogenization (Thermo Fisher Scientific, Waltham, MA, USA).

### Jejunal histomorphology

On day 21, approximately 2 cm mid‐jejunum tissue samples were collected from two randomly selected birds per pen and fixed in 10% buffered formalin. The formalin‐fixed jejunum tissues were dehydrated in graded concentrations of ethanol, 50%, 70% and 96% ethanol for 15 min each, and 100% ethanol for 30 min, cleared with xylene, and embedded in paraffin wax. This was sectioned into 5 μm and stained with haematoxylin‐eosin and the images were captured at 4 × magnification with the aid of BZ microscope (BZ‐X800; Keyence Inc., Itasca, IL, USA) and analyzed using BZ‐X800 Analyzer (Keyence Inc.). Villus height was measured as the distance from the tip of the villus to the crypt junction and the crypt depth as the depth of the invagination between adjacent villi. Six villi and the corresponding crypts were measured per section, and the villus height‐to‐crypt depth ratio was calculated.

### Chemical analysis

The oven‐dried samples of diets, ileal digesta and excreta were ground (0.5 mm) and used for dry matter (DM), nitrogen (N), titanium dioxide and gross energy determination (GE). A portion of the samples was dried at 100 °C for 24 h to evaluate the DM.^13^ The N content of the samples was measured by the combustion method^13^ with a N analyzer (LECO, St Joseph, MI, USA) and the gross energy with the aid of isoperibol bomb calorimeter calibrated with benzoic acid (Model 6200; Parr Instruments, Moline, IL, USA). The titanium dioxide (included as a digestibility marker) content of the samples was measured according to the method of Short *et al* ^14^ The total starch analysis was carried out using a Megazyme assay kit (K‐AMYL; Megazyme, Bray, Ireland). The RS analysis was conducted by Eurofins Food Testing (Heerenveen, The Netherlands), based on AOAC^13^ method 2011.25.

### Quantitative real‐time PCR analysis

On day 21, samples of mid‐jejunal tissue obtained from one randomly selected bird per pen were snap‐frozen in liquid nitrogen and stored at −80 °C prior to RNA extraction. RNA was extracted with QiAzol lysis reagent (Invitrogen, Carlsbad, CA, USA) and purified using RNeasy Mini Kit (Qiagen, Valencia, CA, USA). A high‐capacity cDNA reverse transcription kit (Thermo Fisher Scientific) was used for reverse transcription of RNA to cDNA in a 20‐μL reaction volume, and the RT‐qPCR performed with StepOnePlus (Applied Biosystems, Carlsbad, CA, USA) and Universal SYBR Green Supermix (Bio‐Rad, Hercules, CA, USA). Beta‐actin was used as the housekeeping gene, and the fold change was determined based on 2^−∆∆CT^ method.^15^ The primer sequence is shown in Table 4.

**Table 4:** List of primers and their sequences

Symbol	Gene name	Function	Forward primer	Reverse primer
*β‐actin*	βeta‐actin	Housekeeping gene	ACCGGACTGTTACCAACACC	GACTGCTGCTGACACCTTCA
*MUC‐2*	Mucin‐2	Mucin secretion	ATGCGATGTTAACACAGGACTC	GTGGAGCACAGCAGACTTTG
*GLP‐2*	Glucagon‐like peptide‐2	Cell proliferation	CGTGCCACAGCCATTCTTA	AGCGGCTCTGCAAATGATTA
*PYY*	Peptide tyrosine tyrosine	Satiety regulation	CGCCATTACATCAACCTGGT	GAGTCATCGTCGATGTCGGA
*GLP‐1*	Glucagon‐like peptide‐1	Satiety regulation	GATAGTTCAAGGCAGCTGGC	CTTGAGAGCTGATCCGGGAA
*GLUT‐2*	Glucose transporter‐ 2	Glucose transporter	TCATTGTAGCTGAGCTGTT	CGAAGACAACGAACACATAC
*PepT1*	Peptide transporter‐1	Peptide transporter	AGCCTCAAATGGGATTGGATT	CATCAACTTGCATTCGCTTCA
*y + LAT1*	y + L amino acid transporter 1	Cationic amino acid transporter	CAGAAAACCTCAGAGCTCCCTTT	TGAGTACAGAGCCAGCGCAAT

### Calculations

The index method was used to calculate the apparent ileal digestibility and the total tract nutrient retention of starch, energy and DM. The calculations were conducted on a DM basis. The following equations outline the calculations:

$$\text{Digestibility}\;\left(\%\right)=100\times \left[1-\left(\left(\mathrm{Ci}/\mathrm{Co}\right)\times \left(\mathrm{No}/\mathrm{Ni}\;\right)\right)\right]$$

(1) $$\mathrm{DM}\;\text{Digestibility}\;\left(\%\right)=100\times \left[1-\left(\mathrm{Ci}/\mathrm{Co}\right)\right]$$

where *Ci* and *Co* are the % titanium dioxide concentrations in the diet and excreta/digesta, respectively, and *Ni* and *No* are the percentage nutrient contents in the diet and excreta/digesta, respectively.

Nitrogen‐corrected apparent metabolizable energy (AMEn) and apparent metabolizable energy (AME) in MJ kg^−1^ were calculated by:

$$\mathrm{AME}=\mathrm{GEi}-\left[\left(\mathrm{Ci}/\mathrm{Co}\right)\times \left(\mathrm{GEo}\right)\right]$$

(2) $$\text{AMEn}=\mathrm{AME}-\left[0.0344\times \left(\mathrm{NR}/\mathrm{DMI}\right)\right]$$

where *GEi* and *GEo* represent the diets and excreta gross energy contents (MJ kg^−1^), respectively. The *DMI* (dry matter intake) and *NR* (retained nitrogen) were calculated using:

$$\mathrm{NR}\;\left(g\right)=\left(\mathrm{NI}-\mathrm{NO}\right)$$

(3) $$\mathrm{DMI}\;\left(g\right)=\text{Feed intake}\;\left(\;g\right)\times \text{Feed}\;\mathrm{DM}\left(\text{coefficient}\right)$$

where *NI* and *NO* represent the N intake (g) and output (g), respectively.

### Statistical analysis

The statistical model for the experiment was:

(4) $$Y_{\text{ijkl}}=\mu +\alpha_{i}+\beta_{j}+{\left[\beta \left(\gamma \right)\right]}_{\mathrm{jk}}\;{\left[\beta \left(\delta \right)\right]}_{\mathrm{jl}}+{\left[\beta \left(\gamma \delta \right)\right]}_{\mathrm{jkl}}+\varepsilon_{\text{ijkl}}$$

where *Y*_*ijkl*_ is the response variable, *μ* is the overall mean, α_*i*_ is the random effect of the *i*th block, 𝛽_j_ is the fixed effect of the *j*th treatment group, 𝛽(*γ*)_*jk*_ represent the fixed effect of the *k*th resistant starch level within the *j*th treatment group, 𝛽(*δ*)_*jl*_ represents the fixed effect of the *l*th resistant starch feeding duration within the *j*th treatment group, 𝛽(*γδ*)_*jkl*_ *is the* interaction effect of the *k*th resistant starch level and *l*th resistant starch feeding duration within *j*th treatment group, and *ε*_*ijkl*_ is the residual error. Data obtained were subjected to statistical analysis using the mixed model procedure of JMP Pro 13.2.0 (SAS Institute Inc., Cary, NC, USA) in a 3 × 3 + 1 factorial arrangement. The factors included three levels of resistant starch, each tested across three feeding durations and nested with the control diet. Tukey's honestly significant difference was used to separate means with significant difference. *P* < 0.05 was considered statistically significant. The main effects were reported when no significant interactions are observed, and simple effects discussed in cases where interactions were significant.

## RESULTS

### Growth performance

The growth performance results showed no significant (*P* > 0.05) RSD × RSL and main effects of RSD on WG, FI and BWG at day 21 (Table 5). However, there was a significant main effect of RSL (*P* < 0.05) on FCR. The FCR was lower (*P* < 0.043) in birds receiving 35 g kg^−1^ HCS than in those receiving 25 g kg^−1^ RPS.

**Table 5:** Growth performance response (days 0 to 21) of broiler chickens fed dietary resistant starches with different feeding durations

RSL (g kg^−1^)	RSD (days)	WG (g)	FI (g)	FCR	FBW (g)
Control (0)		1071	1441	1.35 AB	1112
Main effects means of resistant starch feeding duration (RSD)					
	21	1035	1386	1.34	1074
	14	1029	1379	1.34	1070
	7	1015	1362	1.34	1056
Main effects means of resistant starch level (RSL)					
25 RPS		1030	1396	1.36 A	1070
50 RPS		1027	1375	1.34 AB	1067
35 HCS		1023	1356	1.33 B	1064
Pooled SEM		14.5	17.0	0.009	14.6
Simple effect means					
25 RPS	21	1052	1411	1.34	1112
50 RPS	21	1010	1367	1.35	1090
35 HCS	21	1041	1380	1.33	1051
25 RPS	14	1018	1384	1.36	1082
50 RPS	14	1033	1384	1.34	1058
35 HCS	14	1036	1370	1.32	1074
25 RPS	7	1020	1393	1.37	1078
50 RPS	7	1036	1376	1.33	1060
35 HCS	7	990	1319	1.33	1077
Pooled SEM		21.3	24.9	0.013	1031
Probabilities					
RSD		0.529	0.486	0.968	0.557
RSL		0.916	0.159	0.043	0.942
RSD × RSL		0.325	0.574	0.417	0.340

*Note*: Means in a column, within a group, but with different uppercase letters differ significantly (*P* ≤ 0.05). Uppercase letters compare RSL nested with control. *n* = 6 replicate cages for the simple effects and the control, and 18 cages for the main effects of feeding duration and resistant starch level.

RSL, level of the resistant starch source (g kg ^−1^); RSD, duration of feeding the experimental diet (days); RPS, raw potato starch; HCS, high‐amylose corn starch; WG, weight gain; FI, feed intake; FCR, feed conversion ratio; FBW, final body weight.

### Nutrient digestibility, total‐tract nutrient retention and metabolizable energy

There were no significant (*P* > 0.05) RSD × RSL nor main effects of RSD for ileal nutrient digestibility, metabolizable energy (ME), nitrogen‐corrected apparent metabolizable energy (AMEn) and total tract nutrient retention, except for significant (*P* < 0.05) main effects of RSL on apparent ileal digestible energy (AIDE), total tract N retention, ME and AMEn (Table 6). The birds receiving 35 g kg^−1^ HCS had greater (*P* = 0.001) AIDE than those that received 25 g kg^−1^ RPS. Nitrogen retention was greater (*P* = 0.047) in the birds fed the control diet than those fed 50 g kg^−1^ RPS. The birds fed 35 g kg^−1^ HCS had higher (*P* = 0.002) ME and AMEn than those fed the RPS diets.

**Table 6:** Apparent ileal nutrient digestibility (%), total tract nutrient retention (%), and ileal digestible and metabolizable energy for broiler chickens fed dietary resistant starches with different feeding durations

RSL (g kg^−1^)	RSD (days)	Ileal				Total tract				
		DM	N	AIDE (MJ kg^−1^)	Starch	DM	N	ME (MJ kg^−1^)	AMEn (MJ kg^−1^)	Starch
Control (0)		74.7	83.6	13.88^AB^	96.8	71.6	64.0^A^	13.39^AB^	12.94^AB^	96.8
Main effects means of resistant starch feeding duration (RSD)										
	21	70.4	79.6	13.88	95.8	68.7	58.5	13.53	13.03	95.9
	14	70.6	80.1	13.85	95.6	69.2	60	13.65	13.18	96.1
	7	70.2	80.1	13.82	95.5	67.6	58.3	13.38	12.88	95.9
Main effects means of resistant starch level (RSL)										
25 RPS		70.2	80.3	13.58^B^	95.8	68.7	58.8^AB^	3183^B^	12.81^B^	96.2
50 RPS		70.0	79.5	13.79^AB^	95.4	67.1	56.9^B^	3182^B^	12.81^B^	95.5
35 HCS		71.1	80.0	14.2^A^	95.7	69.7	61.1^AB^	3329^A^	13.48^A^	96.2
Pooled SEM		0.691	0.746	0.121	0.199	0.875	1.39	0.152	0.167	0.221
Simple effect means										
25 RPS	21	69.7	79.3	13.50	96.4	68.6	57.6	13.31	12.79	96.4
50 RPS	21	70.1	79.1	13.87	95.4	67.4	56.5	13.28	12.77	95.1
35 HCS	21	71.4	80.4	14.28	95.6	70.1	61.4	13.99	13.54	96.3
25 RPS	14	71.0	80.7	13.71	95.9	68.5	58.9	13.29	12.78	96.1
50 RPS	14	70.6	80.3	13.84	95.2	69.0	60.2	13.66	13.19	95.9
35 HCS	14	70.4	79.3	14.00	95.8	70.0	60.9	14.02	13.56	96.3
25 RPS	7	70.0	80.8	13.56	95.2	68.9	59.9	13.35	12.85	96.3
50 RPS	7	69.2	79.1	13.65	95.7	64.9	54.0	13.01	12.46	95.6
35 HCS	7	71.4	80.3	14.2	95.5	68.9	61.1	13.78	13.33	95.9
Pooled SEM		1.012	1.092	0.180	0.29	1.28	2.03	0.223	0.245	0.383
Probabilities										
RSD		0.874	0.830	0.894	0.403	0.319	0.55	0.324	0.344	0.82
RSL		0.403	0.686	0.001	0.251	0.056	0.047	0.002	0.002	0.055
RSD × RSL		0.705	0.693	0.599	0.068	0.517	0.411	0.608	0.587	0.560

*Note*: *n* = 6 replicate cages for the simple effects and the control, and 18 cages for the main effects of feeding duration and resistant starch level. Means in a column, within a group, but with different uppercase letters differ significantly (*P* ≤ 0.05). Uppercase letters compare RSL nested with control.

RSL, level of the resistant starch source (g kg ^−1^); RSD, duration of feeding the experimental diet (days); RPS, raw potato starch; HCS, high‐amylose corn starch; DM, dry matter; N, nitrogen; AIDE, apparent ileal digestible energy; ME, metabolizable energy; AMEn, nitrogen‐corrected apparent metabolizable energy.

### Caecal content short‐chain fatty acid profile and pH

The caecal SCFA concentrations were not significantly affected by the treatments (Table 7). However, there was a numerical increase in concentration of acetate, propionate, butyrate and total SCFA in birds fed RS diets for 21 days, as well as numerically greater production of acetate, butyrate and total SCFA in birds that received 35 g kg^−1^ HCS diets. In addition, the birds that received the RS diets for 21 days showed a tendency for a lower (*P* = 0.055) caecal content pH than those that received the same diets for 0, 7 or 14 days.

**Table 7:** Quantitative caecal short‐chain fatty acids (SCFA) profile (mM) and caecal content pH of broiler chickens fed dietary resistant starches with different feeding durations

RSL (g kg^−1^)	RSD (days)	Acetate	Propionate	Isobutyrate	Butyrate	Isovalerate	Valerate	Total SCFA	Caecal pH
Control (0)		70.2	4.62	0.64	14.3	0.62	0.95	91.3	7.24
Main effects means of resistant starch feeding duration (RSD)									
	21	77.3	4.36	0.52	17.7	0.50	0.95	101.4	6.92
	14	72.7	4.35	0.51	15.6	0.47	0.99	94.6	7.18
	7	73.4	3.86	0.50	16.3	0.49	0.96	95.5	7.17
Main effects means of resistant starch level (RSL)									
25 RPS		75.1	4.21	0.55	15.8	0.53	0.94	97.1	7.09
50 RPS		71.3	4.21	0.50	16.0	0.48	0.92	93.4	7.06
35 HCS		77.0	4.14	0.47	17.8	0.46	1.03	101.0	7.12
Pooled SEM		2.991	0.348	0.047	1.057	0.054	0.051	3.802	0.096
Simple effect means									
25 RPS	21	79.7	4.46	0.52	16.6	0.48	0.93	102.7	6.96
50 RPS	21	72.1	3.82	0.49	18.2	0.48	0.86	95.9	6.77
35 HCS	21	80.3	4.80	0.55	18.4	0.54	1.07	105.6	7.05
25 RPS	14	76.3	4.39	0.54	15.1	0.47	0.93	97.7	7.14
50 RPS	14	67.8	4.61	0.52	15.1	0.51	1.02	89.6	7.21
35 HCS	14	74.0	4.04	0.46	16.7	0.43	1.01	96.6	7.18
25 RPS	7	69.3	3.78	0.61	15.7	0.63	0.96	91.0	7.17
50 RPS	7	74.1	4.22	0.50	14.7	0.46	0.90	94.9	7.21
35 HCS	7	76.8	3.58	0.40	18.5	0.39	1.02	100.6	7.12
Pooled SEM		4.401	0.512	0.069	1.556	0.080	0.075	5.595	0.141
Probabilities									
RSD		0.377	0.416	0.969	0.250	0.885	0.827	0.278	0.055
RSL		0.288	0.982	0.331	0.235	0.525	0.180	0.278	0.894
RSD ' × RSL		0.564	0.515	0.537	0.845	0.361	0.617	0.803	0.702

RSL, level of the resistant starch source (g kg^−1^); RSD, duration of feeding the experimental diet (days); RPS, Raw potato starch; HCS, High‐amylose corn starch.

*Note*: *n* = 6 replicate cages for the simple effects and the control, and 18 cages for the main effects of feeding duration and resistant starch level.

### Jejunal histomorphology

The RSD × RSL and the main effects of RSD and RSL were not significant for villi height, villi width or villi height:crypt depth ratio (Table 8). However, there was a significant (*P* < 0.05) RSL main effect for the crypt depth; the crypt of birds receiving 25 g kg^−1^ RPS was deeper than for those fed 50 g kg^−1^ RPS.

**Table 8:** Jejunal histomorphology for broiler chickens fed dietary resistant starches with different feeding durations

RSL (g kg^−1^)	RSD (days)	VH (μm)	CD (μm)	VW (μm)	VH:CD
Control (0)		1028	204^B^	185	5.22
Main effects means of resistant starch feeding duration (RSD)					
	21	1042	215	169	5.10
	14	1274	236	167	5.42
	7	1205	235	173	5.25
Main effects means of resistant starch level (RSL)					
25 RPS		1236	251^A^	165	5.01
50 RPS		1187	207^B^	165	5.82
35 HCS		1098	228^AB^	179	4.94
Pooled SEM		127	14.75	5.74	0.474
Simple effect means					
25 RPS	21	1156	248	168	4.88
50 RPS	21	996	169	159	6.03
35 HCS	21	975	227	181	4.38
25 RPS	14	1415	261	157	5.43
50 RPS	14	1311	230	171	5.56
35 HCS	14	1096	219	171	5.27
25 RPS	7	1138	245	169	4.71
50 RPS	7	1254	221	165	5.88
35 HCS	7	1224	238	184	5.16
Pooled SEM		180.6	21.0	8.2	0.675
Probabilities					
RSD		0.241	0.421	0.717	0.700
RSL		0.566	0.035	0.066	0.215
RSD × RSL		0.810	0.495	0.568	0.848

RSL, level of the resistant starch source (g kg^−1^); RSD, duration of feeding the experimental diet (days); RPS, raw potato starch; HCS, high‐amylose corn starch; VH, villi height; CD, crypt depth; VW, villi width.

*Note*: *n* = 6 replicate cages for the simple effects and the control, and 18 cages for the main effects of feeding duration and resistant starch level. Means in a column, within a group, with different uppercase letters differ significantly (*P* ≤ 0.05). Upper case letters compare RSL nested with control.

### Relative mRNA expression of jejunal gut integrity, satiety markers and nutrient transporter gene

The RSD × RSL was significant for relative mRNA expression of jejunal *GLP‐2* (*P* = 0.005) (Table 9), *GLP‐1* (*P* = 0.001) and *GLUT‐2* (*P* = 0.033). The birds fed 25 g kg^−1^ RPS for 21 and 14 days had greater (*P* = 0.005) relative mRNA expression of *GLP‐2* than those fed 25 g kg^−1^ RPS for 7 days. *GLP‐2* also had the highest relative expression in birds. The relative mRNA expression of *GLP‐1* was greater (*P* = 0.001) in birds that received the control diet, 25 or 50 g kg^−1^ RPS, 35 g kg^−1^ HCS for 21 days, and 25 g kg^−1^ RPS for 14 days than in other diets. There was a greater (*P* = 0.033) mRNA relative expression of *GLUT‐*2 for birds fed the control diet, 25 and 50 g kg^−1^ RPS for 21 days, 25 g kg^−1^ RPS for 14 days, or 35 g kg^−1^ HCS for 7 days.

**Table 9:** Relative mRNA expression of jejunal gut integrity gene, satiety hormones, and nutrient transporters in broiler chickens fed dietary resistant starches with different feeding durations

RSL, g kg^−1^	RSD, d	*MUC‐2*	*GLP‐2*	*GLP‐1*	*PYY*	*GLUT‐2*	*PEPT1*	*y + LAT1*
Control (0)		1.00^ab^	1.00^abc^	1.00^a^	1.00^ab,AB^	1.00^a^	1.00	1.00^bc,AB^
Main effects means of resistant starch feeding duration (RSD)								
	21	1.04^a^	1.20	0.91	1.27^a^	0.74	1.41	1.06^b^
	14	1.09^a^	1.34	0.50	1.29^a^	0.70	1.10	1.63^a^
	7	0.68^b^	1.02	0.18	0.72^b^	0.71	1.63	0.50^c^
Main effects means of resistant starch level (RSL)								
25 RPS		0.94	1.24	0.61	1.22^A^	0.78	1.27	1.33^A^
50 RPS		0.80	1.06	0.42	0.74^B^	0.65	1.43	1.06^AB^
35 HCS		1.07	1.19	0.56	1.32^A^	0.72	1.44	0.80^B^
Pooled SEM		0.107	0.083	0.088	0.131	0.074	0.074	0.113
Simple effect means								
25 RPS	21	1.04	1.40^ab^	0.88^ab^	1.23	0.85^abc^	1.00	1.55
50 RPS	21	0.95	1.04^abc^	0.80^ab^	0.94	0.81^abc^	1.63	0.97
35 HCS	21	1.12	0.95^abc^	1.05^a^	1.65	0.56^c^	1.60	0.66
25 RPS	14	1.09	1.53^a^	0.89^ab^	1.70	0.83^abc^	1.37	1.89
50 RPS	14	1.11	1.24^abc^	0.44^bc^	0.89	0.57^c^	0.92	1.74
35 HCS	14	1.07	1.25^abc^	0.16^c^	1.27	0.69^abc^	1.00	1.25
25 RPS	7	0.70	0.79^c^	0.06^c^	0.73	0.65^bc^	1.43	0.55
50 RPS	7	0.32	0.89^bc^	0.02^c^	0.39	0.57^c^	1.73	0.47
35 HCS	7	1.02	1.37^abc^	0.46^bc^	1.03	0.91^ab^	1.73	0.50
Pooled SEM		0.157	0.118	0.128	0.187	0.103	0.103	0.167
Probabilities								
RSD		0.006	0.010	<0.001	0.001	0.838	0.086	<0.001
RSL		0.121	0.175	0.172	0.002	0.321	0.721	0.001
RSD × RSL		0.248	0.005	0.001	0.229	0.033	0.340	0.101

RSL, level of the resistant starch source (g kg^−1^); RSD, duration of feeding the experimental diet (days); RPS, raw potato starch; HCS, high‐amylose corn starch; *MUC‐2*, mucin 2; *GLP‐2*, glucagon‐like peptide 2; *GLP‐1*, glucagon‐like peptide 1; *PYY*, peptide tyrosine tyrosine; *GLUT‐2*, glucose transporter‐ 2; *PEPT1*, peptide transporter‐1; *y + LAT1*, y + L amino acid transporter 1.

*Note*: *n* = 6 replicate cages for the simple effects and the control, and 18 cages for the main effects of feeding duration and resistant starch level. Means in a column, within a group, with different letters differ significantly (*P* ≤ 0.05). Lowercase letters compare the RSD group nested with control, or the simple effect means nested with control (in cases of significant interaction). Uppercase letters compare RSL nested with control.

The main effects of RSD were significant (*P* < 0.05) for *MUC‐2* (mucin‐2), *PYY* (peptide YY), *y + LAT1* (AA transporter). *MUC‐2* (*P* = 0.006) and *PYY* (*P* = 0.001) were more highly expressed in birds fed RS for 21 and 14 days than those fed for 7 days, except the control diet, whereas, *y + LAT1* had greater (*P* < 0.001) relative expression in birds that received RS diet for 14 days than other diets. The main effects of RSL were significant (*P* < 0.05) for *PYY* and *y + LAT1*. *PYY* had higher (*P* = 0.002) relative expression in birds fed 25 g kg^−1^ RPS and 35 g kg^−1^ HCS than 50 g kg^−1^ RPS with the exemption of control diet. The mRNA expression of *y + LAT1* was greater (*P* = 0.001) in birds that received 25 g kg^−1^ RPS than those fed 35 g kg^−1^ HCS.

## DISCUSSION

The potential of RS in improving gut health with little or no detrimental effect on growth performance has been demonstrated in both humans and animals.^16^, ^17^ Continuoussupply of SCFA as a result of RS fermentation in distal part of the digestive tract, especially in animals fed *ad libitum*, may reduce glucose oxidation and lead to no‐detrimental effect or improved growth performance. This varies among RS sources and levels, necessitating the need to determine the optimum source and levels as well as safe feeding duration in various species of animals.^18^, ^19^ On the other hand, the feed milling process can compromise the efficiency of RS with respect to improving gut health by altering the crystalline structure of RS depending on the stability to the shearing process and pelleting temperature.^20^, ^21^ In the preceding experiment, we investigated the impact of different types and inclusion of RS on growth performance, gut morphology and gut health metrics.^6^ The RS types and inclusion levels used in the current experiment were informed by its predecessor. The experiment length was limited to the starter and early grower phases as a result of facility limitations because the birds could not be kept in metabolism cages beyond 21 days. The 3‐week duration of the experiment was conveniently divided into three 1‐week phases for practical purposes. The current experiment investigated how different durations of the feeding of raw potato starch (RPS) and high amylose corn starch (HCS) could influence their effects on the growth performance, nutrient utilization, intestinal morphology, and cecal SCFA profile in broiler chickens during the grower phase. It was hypothesized that the varied impacts of dietary RS levels on the production of caecal SCFA, nutrient utilization, intestinal morphology, modulation of mRNA expression of jejunal nutrient transporters and genes of gut health importance depend on the duration of feeding RS in broiler chickens.

### Growth performance

The results obtained on growth performance in this study showed no detrimental effect of RS on growth performance metrics, which could be because of the relatively short duration of feeding RS diets, and the lower FCR observed in the birds fed 35 g kg^−1^ HCS diet relative to the 25 g kg^−1^ RPS diet might be the result of its greater resistance to pancreatic amylase because of its higher amylose content and its potential to form RS3, enabling HCS to maintain most of its granular integrity during processing.^22^, ^23^ Generally, the non‐significant effects of RS levels and feeding duration on growth performance in the present study are in line with the previous studies that reported no significant effects of graded levels of RPS on growth performance in ducks fed for varied durations, up to 35 days^8^ and in growing pigs fed RS diets.^24^ The difference in the present study compared to that of Qin *et al*.^8^ is the feeding of a standard diet until the experimental diets with RS were introduced. However, some studies reported detrimental effects of RS on growth in animals, attributing this to the low caloric density of RS and variability in the crystalline configuration of different RS.^10^, ^16^, ^17^, ^25^ This is especially because Liu *et al*.^10^ suggested that high crystallinity of resistant starch (RS) may reduce starch digestibility and limit energy intake, leading to depressed growth in broiler chickens. Additional probable reasons for variable effects of RS on growth performance, aside from the source of RS and age, might be a result of differences in the presentation of diets, either as mash or pellets influencing the viability of the crystalline structure, a high concentration of RS in diets, or species differences, amongst other factors.^2^, ^20^, ^21^, ^23^

### Nutrient digestibility, total‐tract nutrient retention and metabolizable energy

Greater AIDE, ME and AMEn in birds fed 35 g kg^−1^ HCS than all RPS and control diets indicated improved energy utilization in HCS diets than other RS diets irrespective of the feeding duration. The greater DM and starch retention in the birds that received control diet was not unexpected because the RS diet has been shown to reduce starch digestibility.^19^, ^26^ These results along with our previous findings indicated that HCS may be a preferrable RS for broiler chickens, among those tested in our experiments. Other studies observed lower total tract nutrient utilization and apparent ileal digestibility of nutrients with higher dietary levels of RS^23^, ^27^ but a previous study by Oluseyifunmi *et al*.^19^ observed an increase in N retention with increasing RPS and HCS levels and higher AME and AMEn in birds fed 100 g kg^−1^ HCS diet than in banana starch and raw potato starch in broiler chickens. Liu *et al*.^10^ observed a linear decrease in the apparent retention of DM and total starch with increasing RS levels from days 18 to 20 and days 39 to 41 in Arbor acre broiler chickens fed dietary 40, 80 and 100 g kg^−1^ Hi‐maize resistant starch. Higher energy digestibility and availability in birds fed 35 g kg^−1^ HCS explains the lower FCR observed at this level of inclusion, although the FI and WG were not significantly different across all the diets. Sources of RS play a fundamental role in determining the crystalline organization of RS, which is pertinent to its amylolytic hydrolysis^21^, and the effects of RS on nutrient digestibility and growth performance in broiler chicken may be more dependent on the amount ingested rather than on the feeding duration of the RS used in this study.

### Caecal content short‐chain fatty acid profile and pH

SCFAs are the major beneficial end‐products of the distal gastrointestinal tract microbial fermentation of dietary fibers, which constitute the fundamental mode of action of RS in improving gut health in animals. The result obtained on caecal SCFA concentrations in the present study showed no significant effects of the treatments, However, acetate, butyrate and total SCFA were numerically greater in birds fed 35 g kg^−1^ HCS and those fed RS diets for 21 days. Previous findings showed significant increase in SCFA as a result of increased RS fermentation in the caeca of birds fed dietary RS.^19^, ^28^, ^29^ The probable explanation for the difference in the results could be the possible alteration of the crystalline structure of RS by feed milling processes involved in pelleting the diets^21^ because the diets fed in our previous study were presented as mash through starter to grower phases, whereas, in the present study, the diets were in mash form in the starter phase and as pellets in the grower phase. The possibility of alteration in RS crystalline structure cannot be ruled out during feed processing, which could alter their physiochemical properties and fermentability.^21^, ^30^ Lower luminal and digesta pH are associated with the mechanism of action of SCFA with respect to modulating intestinal microbiota, which could favor the proliferation of beneficial bacteria relative to pathogenic ones.^31^ The tendency for lower caecal content pH in birds fed RS diets for a longer period (21 days) is evidence of RS fermentation and increased SCFA production in the birds fed RS diets for a longer duration.^2^

### Jejunal histomorphology

The development and absorptive capacity of the intestine is measured by the villi height to crypt depth ratio.^32^ The result observed for jejunal histomorphology showed that neither the duration of feeding the RS diets, nor dietary levels of RS in the diets influenced the villi height, width or villi height: crypt depth ratio except for deeper crypt in birds fed the 25 g kg^−1^ RPS diet. This observation is consistent with our previous findings.^19^

By contrast, Qin *et al*.^8^ reported longer villi height, higher villi height:crypt depth ratio and lower crypt depth in the ileum of ducks fed 120 and 180 g kg^−1^ RPS diets relative to 0 g kg^−1^ RPS diet for 14 and 35 days and Liu *et al*.^10^ observed a decrease in the jejunal villi height and villi height:crypt depth ratio of broiler chickens fed diets containing 110.2 and 141.6 g kg^−1^ or 114.8 and 147.8 g kg^−1^ Hi‐maize resistant starch, in the starter and grower phases, respectively, each for 21 or 42 days. However, differences in the RS inclusion level, intestinal site and species are the probable explanation for disparity in these results, especially because the major site of fermentation of RS lies in the distal part of the intestine.

### Relative mRNA expression of jejunal gut integrity, satiety markers and nutrient transporters genes

Resistant starches, especially high‐amylose corn starch, have been linked with increased fermentation, and elevated expression of *GLP‐1* (glucagon‐like peptide‐1) and *PYY* (peptide YY) genes in the distal GIT, as well as improved gut health.^33^, ^34^ GLP‐1 and PYY are satiety hormones associated with feed intake regulation.^34^ Glucagon‐like peptide‐2 (*GLP‐2*) is produced along with *GLP‐1*, and it is associated with increased crypt cell proliferation and nutrient absorption in pigs and humans.^35^

The increased mRNA expression of *GLP‐2* by longer feeding of 25 g kg^−1^ RPS for a period of 14 and 21 days corresponded with the deeper crypt observed in birds fed at this level and a pointer to the ability of *GLP‐2* with respect to increasing crypt proliferation. This was reflected by the greater FCR in birds fed 25 g kg^−1^ RPS but showed no negative implication on the growth performance, probably because the levels of expression of *GLP‐1* and *PYY* (satiety hormones) in RS‐fed birds were below the concentration capable of decreasing feed intake in that they were similar to the expression observed in the birds that received the control diet.

MUC‐2 codes for mucin which lubricates the intestinal mucosa, functions in protecting the gut and maintaining intestinal homeostasis. It constitutes a part of the first line of defense against microbial invasion.^36^, ^37^ In the present study, the relative mRNA expression of *MUC‐2* was elevated in birds that received RS for 14 and 21 days. Similarly, feeding 180 g kg^−1^ RPS diet in meat‐type ducks for 14 days elevated *MUC‐2* mRNA expression in the ileum^8^ reported a greater caecal *MUC‐2* mRNA expression in the pigs fed dietary inclusion of 50 g kg^−1^ RPS. Our result demonstrated the duration‐dependent effects of dietary RS on jejunal *MUC‐2* expression in broiler chickens. Feeding RS diets for 7 days was not sufficiently long enough time to elicit a response except when fed for a longer period, as observed in the birds that received RS diets for 14 or 21 days.

GLUT‐2 is a facilitative glucose transporter that allows large bidirectional fluxes of glucose in and out the cell.^38^ The variability of mRNA expression of *GLUT‐2* at different levels and durations of feeding RS indicated the interdependence of both factors in modulation of luminal glucose uptake.^39^ Furthermore, the major role of *y + LAT1* (Na‐dependent cationic amino acid exchanger) is to transport amino acids across the intestinal epithelium.^40^, ^41^ The greater expression of y+LAT1 in birds fed RS diet for a period of 14 d and at 25 g kg‐1 RPS level than 35 g kg‐1 HCS further demonstrated the importance of both factors in nitrogen uptake during RS consumption in broiler chickens.^19^, ^42^ The latter along with greater nitrogen retention at lower RS concentrations further explained the reason why the increase in crypt proliferation (evidenced by increase in crypt depth at 25 g kg^−1^ RPS inclusion) had no negative effect on the weight gain and final body weight. The improved nitrogen intake and utilization contributed to the growth of these animals, which might have compensated for the endogenous nitrogen loss associated with increased enterocyte turnover rate because deeper crypts are suggestive of a higher enterocyte turnover rate,^43^, ^44^

## Conclusions

The present study assessed the impacts of feeding graded levels (RSL) of raw potato starch (RPS) and high amylose corn starch (HCS) on growth performance, nutrient utilization, intestinal morphology and cecal SCFA profile in broiler chickens as related to feeding duration or duration of adaption to RS diets. The results showed no significant effects of RSD on growth performance or nutrient digestibility. The RSL influenced the FCR, AIDE, ME and AMEn, with 35 g kg^−1^ HCS showing better efficiency than 25 g kg^−1^ RPS diets and greater AIDE, AME and AMEn in birds fed 35 g kg^−1^ HCS diets. The mRNA expression of genes was influenced by both RSD and RSL, with notable increases in the expression of certain markers of gut integrity and nutrient transporters such as *GLP‐2*, *MUC‐*2 and *y + LAT1*. Within the context of the current experiment (3 weeks in duration), the lack of translation of the RSD effect to the growth performance response indicates that the latter was mainly influenced by the amount of RS in the diet rather than how long the birds were fed such diets. However, it would be pertinent for future studies to investigate the effects of the feed milling process on the crystalline integrity of RS, resistance to pancreatic amylase and the efficiency of microbial fermentation of RS when animals are fed pelletized diets.

## CONFLICTS OF INTEREST

The authors declare that they have no conflicts of interest.

## AUTHOR CONTRIBUTIONS

OAO was responsible for conceptualization, acquisition of funds, validation, reviewing and editing. IWO was responsible for conceptualization, laboratory work, data analysis, original draft preparation and editing.

## FUNDING

This study was sponsored by cooperative agreement 58‐6040‐8‐034 (United States Department of Agriculture) – Agricultural Research Service.

## References

[1] Delcour JA and Hoseney RC , Principles of Cereal Science and Technology. AACC International, St. Paul, Minnesota (2010).

[2] Tan FPY , Beltranena E and Zijlstra RT , Resistant starch: Implications of dietary inclusion on gut health and growth in pigs: a review. J Anim Sci Biotechnol 12:124 (2021). 34784962 10.1186/s40104-021-00644-5 PMC8597317

[3] Choct M , Hughes RJ and Bedford M , Effects of a xylanase on individual bird variation, starch digestion throughout the intestine, and ileal and caecal volatile fatty acid production in chickens fed wheat. Br Poult Sci 40:419–422 (1999). 10475642 10.1080/00071669987548

[4] Dittoe DK , Olson EG and Ricke SC , Impact of the gastrointestinal microbiome and fermentation metabolites on broiler performance. Poult Sci 101:101786 (2022). 35346496 10.1016/j.psj.2022.101786 PMC9079343

[5] Champ MM , Physiological aspects of resistant starch and in vivo measurements. J AOAC Int 87:749–755 (2004). 10.1093/jaoac/87.3.749. 15287675

[6] Oluseyifunmi IW , Lourenco J and Olukosi OA , The interactivity of sources and dietary levels of resistant starches – impact on growth performance, starch, and nutrient digestibility, digesta oligosaccharides profile, cecal microbial metabolites, and indicators of gut health in broiler chickens. Poult Sci 103:104337 (2024). 39388980 10.1016/j.psj.2024.104337 PMC11752116

[7] Haenen D , Zhang J , da Silva CS , Bosch G , van der Meer IM , van Arkel J *et al*., A diet high in resistant starch modulates microbiota composition, SCFA concentrations, and gene expression in pig intestine. J Nutr 143:274–283 (2013). 10.3945/jn.112.169672. 23325922

[8] Qin S , Zhang K , Ding X , Bai S , Wang J and Zeng Q , Effect of dietary graded resistant potato starch levels on growth performance, plasma cytokines concentration, and intestinal health in meat ducks. Poult Sci 98:3523–3532 (2019). 31329991 10.3382/ps/pez186

[9] Nofrarías M , Martínez‐Puig D , Pujols J , Majó N and Pérez JF , Long‐term intake of resistant starch improves colonic mucosal integrity and reduces gut apoptosis and blood immune cells. Nutrition 23:861–870 (2007). 17936196 10.1016/j.nut.2007.08.016

[10] Liu Y , Zhang Y , Li J , Wang X , Xing T , Zhu X *et al*., Growth performance, carcass traits and digestive function of broiler chickens fed diets with graded levels of corn resistant starch. Br Poult Sci 61:146–155 (2020). 31735080 10.1080/00071668.2019.1694137

[11] Cobb‐Vantress , Cobb500 Broiler Performance and Nutrition Supplement https://www.cobb-vantress.com.

[12] Lourenco JM , Kieran TJ , Seidel DS , Glenn TC , Silveira MF , Callaway TR *et al*., Comparison of the ruminal and fecal microbiotas in beef calves supplemented or not with concentrate. PLoS One 15:e0231533 (2020). 10.1371/journal.pone.0231533. 32282837 PMC7153887

[13] AOAC , Official methods of analysis. Association of Official Analytical Chemists, Washington, DC (2016).

[14] Short F , Gorton P , Wiseman J and Boorman K , Determination of titanium dioxide added as an inert marker in chicken digestibility studies. Anim Feed Sci Technol 59:215–221 (1996).

[15] Livak KJ and Schmittgen TD , Analysis of relative gene expression data using real‐time quantitative PCR and the 2^−ΔΔCT^ method. Methods 25:402–408 (2001). 11846609 10.1006/meth.2001.1262

[16] Li T , Huang R , Wu G , Lin Y , Jiang Z , Kong X *et al*., Growth performance and nitrogen metabolism in weaned pigs fed diets containing different sources of starch. Livest Sci 109:73–76 (2007).

[17] Regmi PR , Metzler‐Zebeli BU , Gänzle MG , van Kempen TA and Zijlstra RT , Starch with high amylose content and low in vitro digestibility increases intestinal nutrient flow and microbial fermentation and selectively promotes bifidobacteria in pigs. J Nutr 141:1273–1280 (2011). 10.3945/jn.111.140509. 21628635

[18] van Erp R , De Vries S , Van Kempen T , Den Hartog L and Gerrits W , Feed intake patterns nor growth rates of pigs are affected by dietary resistant starch, despite marked differences in digestion. Animal 14:1402–1412 (2020). 31852553 10.1017/S1751731119002945

[19] Oluseyifunmi IW , Lourenco J and Olukosi OA , The interactivity of sources and dietary levels of resistant starches–impact on growth performance, starch, and nutrient digestibility, digesta oligosaccharides profile, caecal microbial metabolites, and indicators of gut health in broiler chickens. Poult Sci 103:104337 (2024). 10.1016/j.psj.2024.104337. 39388980 PMC11752116

[20] Devi A , Fibrianto K , Torley P and Bhandari B , Physical properties of cryomilled rice starch. J Cereal Sci 49:278–284 (2009).

[21] Alsaffar AA , Effect of food processing on the resistant starch content of cereals and cereal products–a review. Int J Food Sci Technol 46:455–462 (2011).

[22] Morell MK , Konik‐Rose C , Ahmed R , Li Z and Rahman S , Synthesis of resistant starches in plants. J AOAC Int 87:740–748 (2004). 15287674

[23] Giuberti G , Gallo A , Moschini M and Masoero F , New insight into the role of resistant starch in pig nutrition. Anim Feed Sci Technol 201:1–13 (2015).

[24] Pluske JR , Durmic Z , Pethick DW , Mullan BP and Hampson DJ , Confirmation of the role of rapidly fermentable carbohydrates in the expression of swine dysentery in pigs after experimental infection. J Nutr 128:1737–1744 (1998). 9772144 10.1093/jn/128.10.1737

[25] Higgins JA , Resistant starch and energy balance: impact on weight loss and maintenance. Crit Rev Food Sci Nutr 54:1158–1166 (2014). 24499148 10.1080/10408398.2011.629352 PMC4220782

[26] Morel PC , Melai J , Eady S and Coles G , Effect of non‐starch polysaccharides and resistant starch on mucin secretion and endogenous amino acid losses in pigs. Asian Australas J Anim Sci 18:1634–1641 (2005).

[27] Cervantes‐Pahm SK , Liu Y and Stein HH , Comparative digestibility of energy and nutrients and fermentability of dietary fiber in eight cereal grains fed to pigs. J Sci Food Agric 94:841–849 (2014). 23893839 10.1002/jsfa.6316

[28] Qin S , Zhang K , Applegate TJ , Ding X , Bai S , Luo Y *et al*., Dietary administration of resistant starch improved caecal barrier function by enhancing intestinal morphology and modulating microbiota composition in meat duck. Br J Nutr 123:172–181 (2020). 31495347 10.1017/S0007114519002319

[29] Zhang Y , Liu Y , Li J , Xing T , Jiang Y , Zhang L *et al*., Dietary corn resistant starch regulates intestinal morphology and barrier functions by activating the Notch signaling pathway of broilers. Asian Australas J Anim Sci 33:2008–2020 (2020). 32164060 10.5713/ajas.19.0967 PMC7649406

[30] Svihus B , Uhlen AK and Harstad OM , Effect of starch granule structure, associated components and processing on nutritive value of cereal starch: A review. Anim Feed Sci Technol 122:303–320 (2005).

[31] Regassa A and Nyachoti CM , Application of resistant starch in swine and poultry diets with particular reference to gut health and function. Anim Nutr 4:305–310 (2018). 30175259 10.1016/j.aninu.2018.04.001 PMC6116817

[32] Marchewka J , Sztandarski P , Zdanowska‐Sąsiadek Ż , Adamek‐Urbańska D , Damaziak K , Wojciechowski F *et al*., Gastrointestinal tract morphometrics and content of commercial and indigenous chicken breeds with differing ranging profiles. Animals 11:1881 (2021). 34202789 10.3390/ani11071881 PMC8300197

[33] Zhou J , Martin RJ , Tulley RT , Raggio AM , McCutcheon KL , Shen L *et al*., Dietary resistant starch upregulates total GLP‐1 and PYY in a sustained day‐long manner through fermentation in rodents. Am J Physiol Endocrinol Metab 295:E1160–E1166 (2008). 18796545 10.1152/ajpendo.90637.2008 PMC2584810

[34] Keenan MJ , Martin RJ , Raggio AM , McCutcheon KL , Brown IL , Birkett A *et al*., High‐amylose resistant starch increases hormones and improves structure and function of the gastrointestinal tract: a microarray study. Lifestyle Genomics 5:26–44 (2012). 10.1159/000335319 PMC4030412 22516953

[35] Hellmich MR and Evers BM , in Physiology of the Gastrointestinal Tract, ed. by Johnson LR . Elsevier Academic Press, Amsterdam‐Boston‐Heildelberg, pp. 435–458 (2006).

[36] Cornick S , Tawiah A and Chadee K , Roles and regulation of the mucus barrier in the gut. Tissue Barriers 3:e982426 (2015). 25838985 10.4161/21688370.2014.982426 PMC4372027

[37] Liu Y , Yu X , Zhao J , Zhang H , Zhai Q and Chen W , The role of MUC2 mucin in intestinal homeostasis and the impact of dietary components on MUC2 expression. Int J Biol Macromol 164:884–891 (2020). 32707285 10.1016/j.ijbiomac.2020.07.191

[38] Leturque A , Brot‐Laroche E and Le Gall M , GLUT2 mutations, translocation, and receptor function in diet sugar managing. Am J Physiol Endocrinol Metab 296:E985–E992 (2009). 19223655 10.1152/ajpendo.00004.2009

[39] Sajilata MG , Singhal RS and Kulkarni PR , Resistant starch–a review. Compr Rev Food Sci Food Saf 5:1–17 (2006). 33412740 10.1111/j.1541-4337.2006.tb00076.x

[40] Wang C‐Y , Liu S , Xie X‐N and Tan Z‐R , Regulation profile of the intestinal peptide transporter 1 (PepT1). Drug Des Devel Ther 11:3511–3517 (2017). 10.2147/DDDT.S151725. PMC5726373 29263649

[41] Lin Y , Teng P‐Y and Olukosi OA , The effects of xylo‐oligosaccharides on regulating growth performance, nutrient utilization, gene expression of tight junctions, nutrient transporters, and cecal short chain fatty acids profile in Eimeria‐challenged broiler chickens. Poult Sci 101:102125 (2022). 36088820 10.1016/j.psj.2022.102125 PMC9468463

[42] Lin Y , Lourenco JM and Olukosi OA , The effects of protease, xylanase, and xylo‐oligosaccharides on growth performance, nutrient utilization, short‐chain fatty acids, and microbiota in Eimeria‐challenged broiler chickens fed low‐protein diet. Poult Sci 102:102789 (2023). 37354614 10.1016/j.psj.2023.102789 PMC10404748

[43] Montagne L , Pluske J and Hampson D , A review of interactions between dietary fibre and the intestinal mucosa, and their consequences on digestive health in young non‐ruminant animals. Anim Feed Sci Technol 108:95–117 (2003).

[44] Wijtten P , Langhout D and Verstegen M , Small intestine development in chicks after hatch and in pigs around the time of weaning and its relation with nutrition: A review. Acta Agric Scand A Anim Sci 62:1–12 (2012).

